# Blood Changes in Disease

**Published:** 1853-01

**Authors:** 


*Blood Changes in Disease.*—A recent elaborate memoir on diseases of the blood. presented to the Academy of Sciences by MM. Becquerel and Rodier, winds up with the following conclusions:

1. In most chronic diseases, or in consequence of various hygienic influences, the three principal elements of the blood, viz: the red particles, the fibrin, and the albumen, may diminish or augment in quantity, separately or conjointly.

2. The red particles diminish in most prolonged chronic diseases, especially in organic diseases of the heart, in Bright's disease, chlorosis, paludal cachexia, hemorrhages, the last period of tuberculosis, the cancerous diathesis; the globules diminish also when the individual has been insufficiently nourished or exposed to bad hygienic conditions, such as insufficiency of air, want of light, or to humidity.

3. The albumen diminishes in the last stage of heart diseases, great symptomatic anæmia, cancerous diathesis, and when the nourishment is insufficient.

4. The fibrin remains normal, or is sometimes augmented in acute scurvy. In chronic scurvy, and in the symptomatic scorbutic state, which attends some chronic maladies, especially in organic diseases of the heart, it diminishes.

5. In all the above cited cases the water of the blood increases in amount.

6. The diminution of globules is shown especially by decoloration of the skin, palpitation, dyspnœa, systolic murmur at the base of the heart, arterial and venal murmurs.

7. The diminution of albumen produces dropsy; speaking generally, dropsey is the symptom of diminution of albumen.

8. The diminution of fibrin shows itself by cutaneous and mucous hemorrhages.

9. In the symptomatic anæmia of great hemorrhages, of bad nourishment, and of too great catamenial flow, the specific gravity of the blood diminishes, the water augments, the blood globules diminish; the albumen remains normal, or slightly diminishes; the fibrin remains the same.

10. In chlorosis, which is an entirely different disease from anæmia, the blood may be completely normal. When any changes occur, they are in the augmentation of the water, the diminution of the red particles, and the conservation of its normal figure or the augmentation of the fibrin.

11. In acute Bright's disease, the fibrin remains the same, the albumen diminishes; in chronic Bright's disease, the globules and albumen diminish; the fibrin remains normal or diminishes.

12. Most dropsies, called essential, are due to a diminution of the albumen.

13. In heart disease, the blood deteriorates more and more as the disease advances. The changes consist in simultaneous diminution of the red globules, albumen, and fibrin.

14. In acute scurvy, the blood is not altered; in chronic scurvy the fibrin is diminished, and the globules augmented.

15. Quinine and generous diet are the best treatment for diminution of the albumen; vegetable acids and good diet for diminution of the fibrin; iron for the diminution of the red particles.—*Phil. Med. Jour.*

